# Supplementary material for: Investigation of Thermodynamic Properties of Dimethyl Phosphate-Based ILs for Use as Working Fluids in Absorption Refrigeration Technology
Source: Molecules. 2023 Feb 17;28(4):1940. doi: 10.3390/molecules28041940 (PMC9961247; doi:10.3390/molecules28041940)
Supplement: Supplementary file 1 [file molecules-28-01940-s001.zip › molecules-2219091-supplementary.pdf]

Supplementary Material

Investigation of Thermodynamic Properties of Dimethyl Phosphate-Based ILs for Use as Working Fluids in Absorption Refrigeration Technology

Michał Skonieczny [1,*], Marta Królikowska [2] and Marek Królikowski [2,*]

[1] Doctoral School, Warsaw University of Technology, Plac Politechniki 1, 00-661 Warsaw, Poland
[2] Department of Physical Chemistry, Faculty of Chemistry, Warsaw University of Technology, Noakowskiego 3, 00-664 Warsaw, Poland; marta.krolikowska@pw.edu.pl
* Correspondence: michal.skonieczny.dokt@pw.edu.pl (M.S.); marek.krolikowski@pw.edu.pl (M.K.)

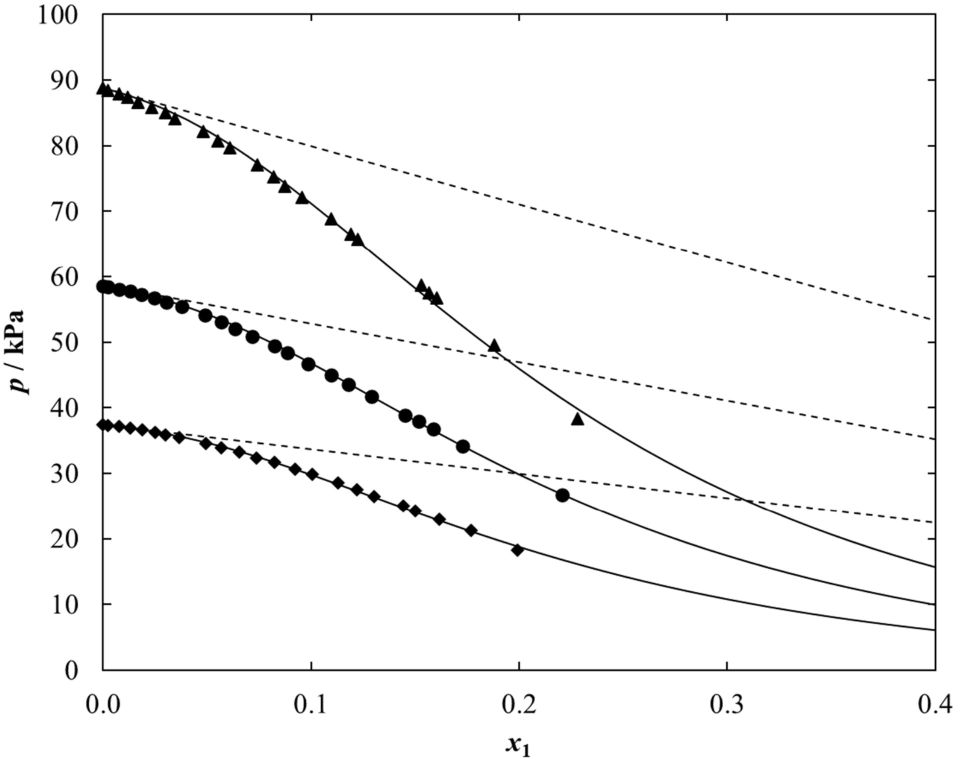

Figure S1. Plot of the Experimental and Calculated *P*–*x* Data for {[C_1C_2PIP][DMP] (1) + Ethanol (2)} *vs*. Ionic Liquid Mole Fraction, x_1, at Different Temperatures, *T*: ♦, 328.15 K; ●, 338.15 K; ▲, 348.15 K. Full Points—Experimental Data; Solid Lines—NRTL Equation With Parameters Given in Table 7; Dashed Lines—Ideal Solution.

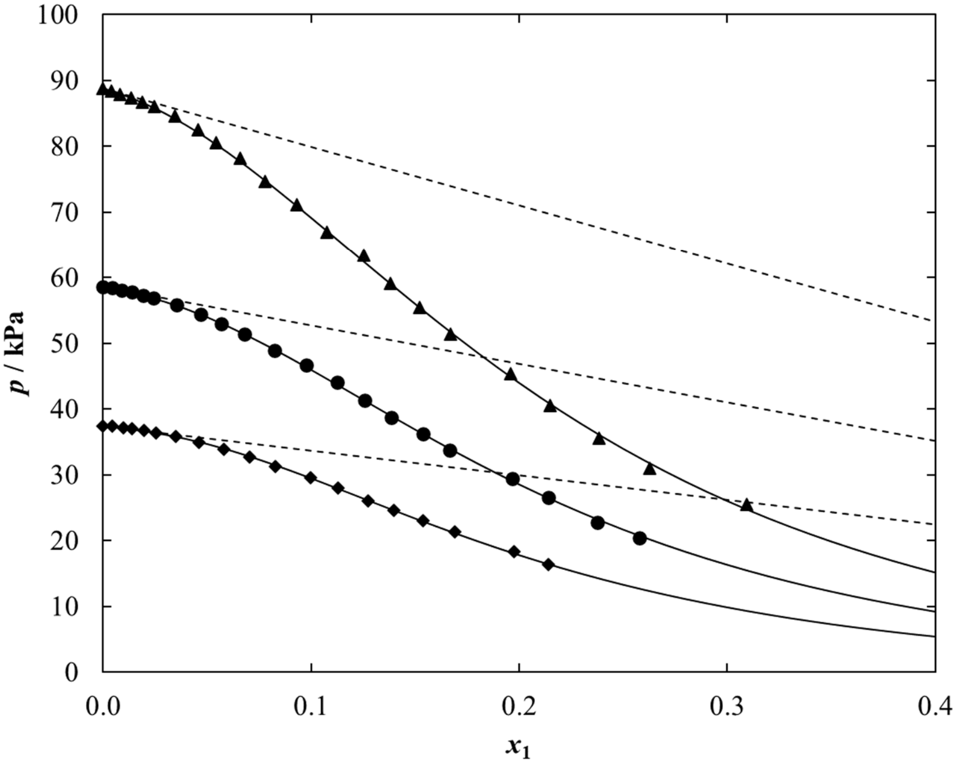

Figure S2. Plot of the Experimental and Calculated *P*–*x* Data for {[$N_{1,2,2,2}$][DMP] (1) + Ethanol (2)} *vs*. Ionic Liquid Mole Fraction, x_1, at Different Temperatures, *T*: ♦, 328.15 K; ●, 338.15 K; ▲, 348.15 K. Full Points—Experimental Data; Solid Lines—NRTL Equation With Parameters Given in Table 7; Dashed Lines—Ideal Solution.

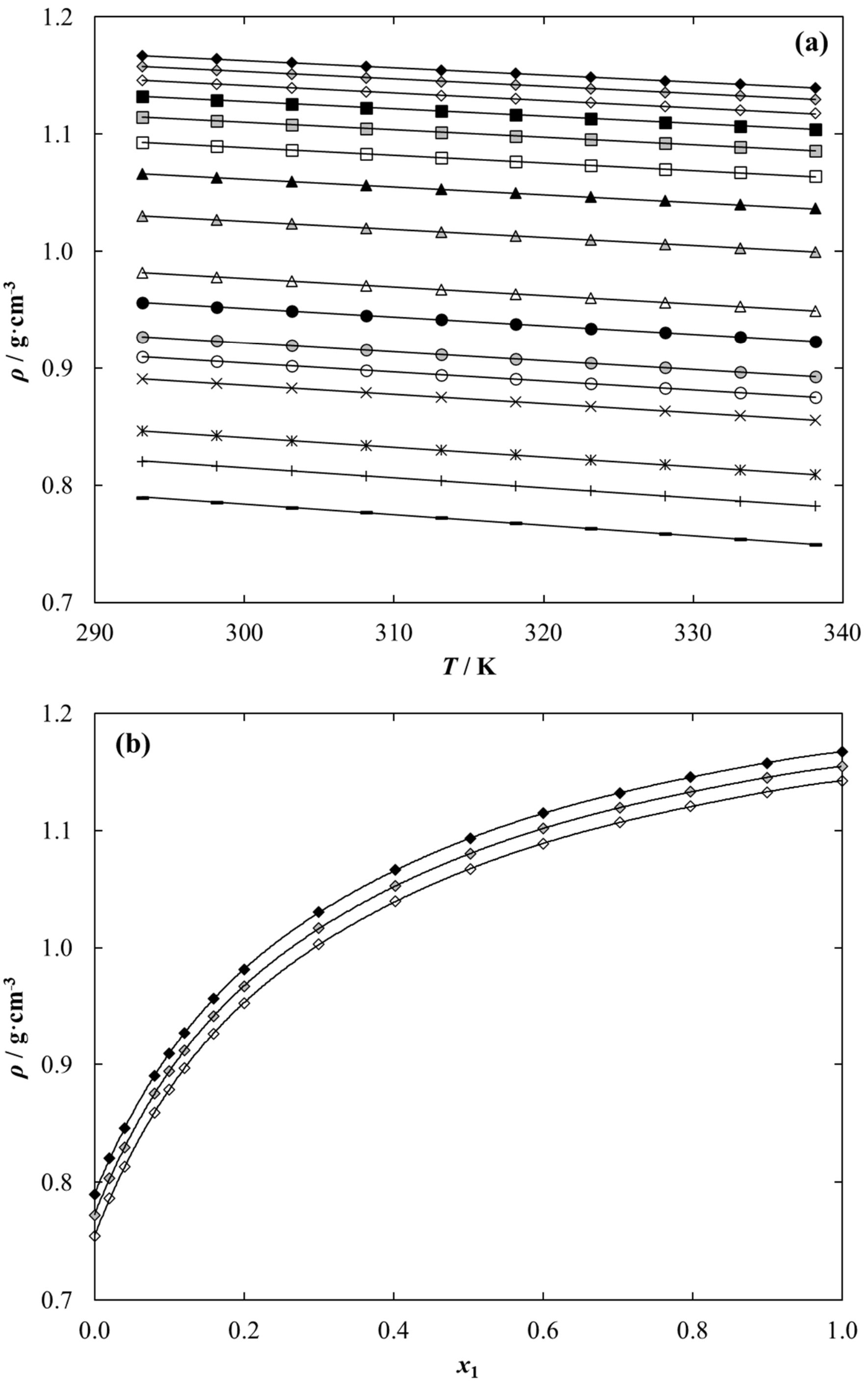

Figure S3. Temperature and Composition Dependence of Liquid Density Data for {[C_1C_2PIP] [DMP] (1) + Ethanol (2)} system as a function of **(a)** temperature for different composition, x_1: ♦, 1.0000; ♦, 0.8993; ◊, 0.7968; ■, 0.7031; ■, 0.6007; □, 0.5028; ▲, 0.4027; ▲, 0.3001; Δ, 0.2001; ●, 0.1597; ●, 0.1203; ○, 0.1001; x, 0.0803; *, 0.0402; +, 0.0203; –, 0.0000; **(b)** composition at different temperature, T: ♦, 293.15 K; ◊, 303.15 K; ●, 313.15 K; ○, 323.15 K; ▲, 333.15 K. Points—Experimental Data; Solid Lines—Correlation Using Eq. (10) With Parameters Given in Table 11.

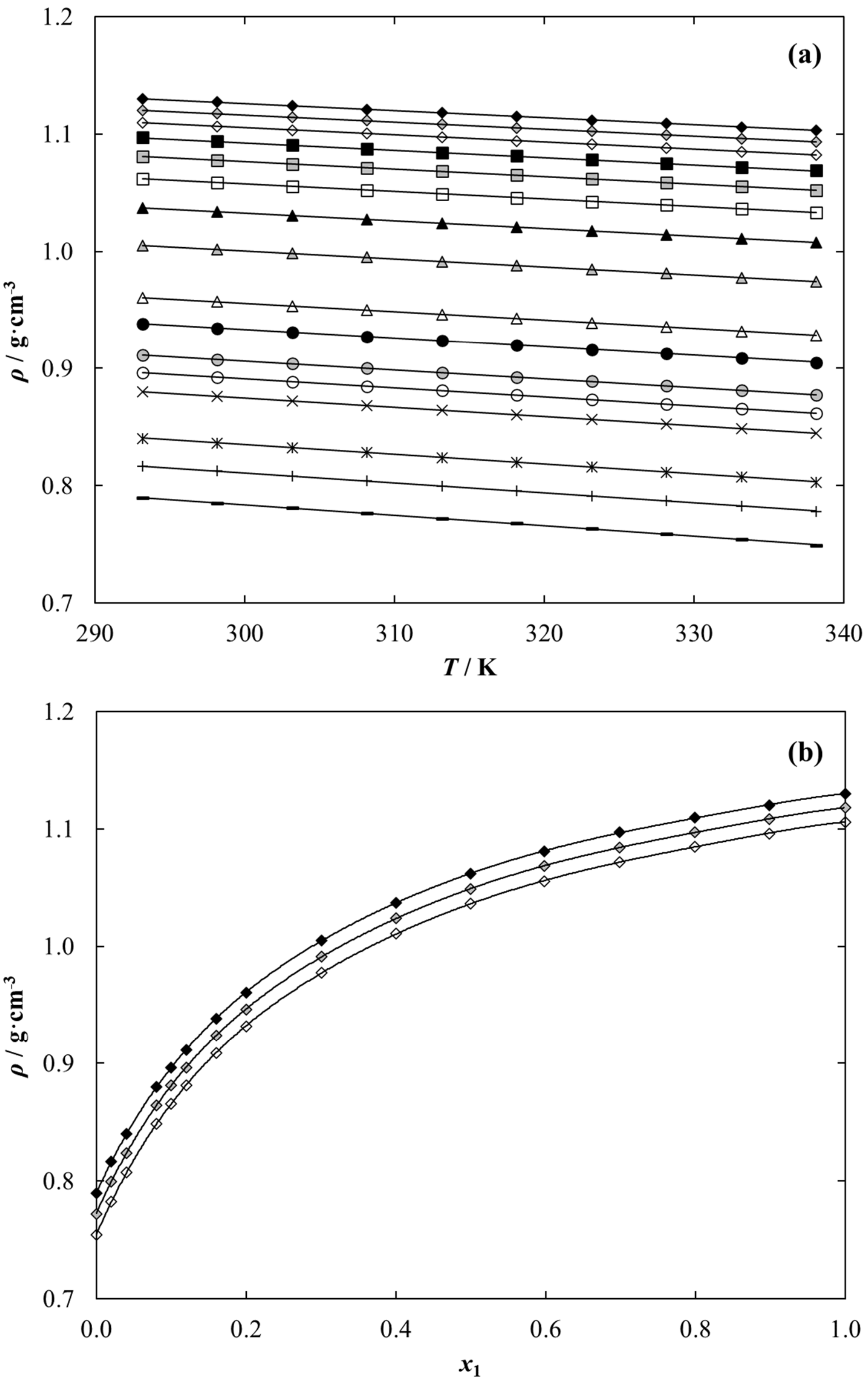

Figure S4. Temperature and Composition Dependence of Liquid Density Data for {$[N_{1,2,2,2}]$ [DMP] (1) + Ethanol (2)} system as a function of **(a)** temperature for different composition, x_1: ♦, 1.0000; ♦, 0.8987; ◊, 0.7991; ■, 0.6988; ■, 0.5986; □, 0.4998; ▲, 0.4004; ▲, 0.3011; Δ, 0.1999; ●, 0.1600; ●, 0.1201; ○, 0.0998; x, 0.0802; *, 0.0402; +, 0.0201; –, 0.0000; **(b)** composition at different temperature, T: ♦, 293.15 K; ◊, 303.15 K; ●, 313.15 K; ○, 323.15 K; ▲, 333.15 K. Points—Experimental Data; Solid Lines—Correlation Using Eq. (10) With Parameters Given in Table 11.

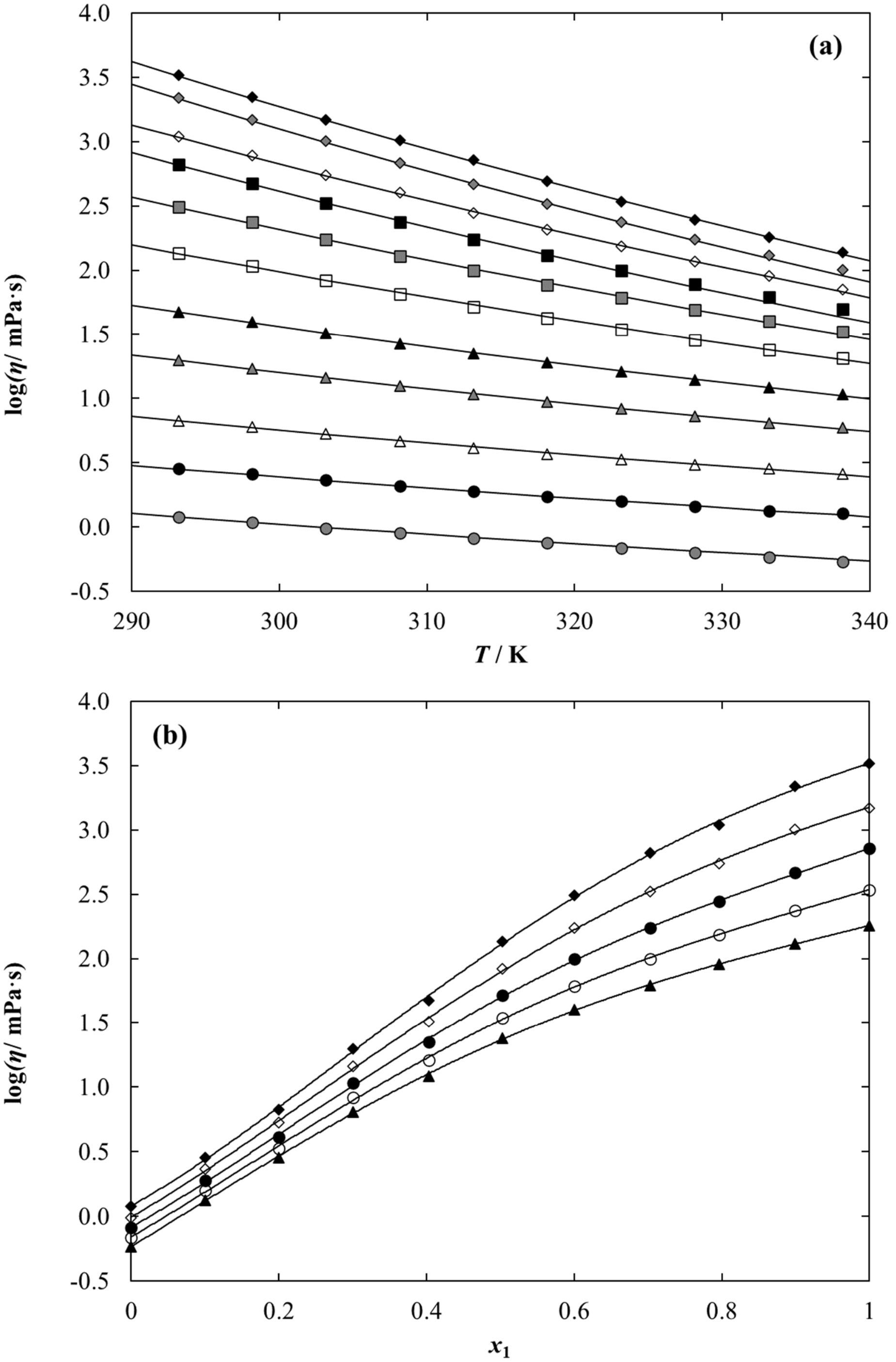

Figure S5. Experimental and Calculated Dynamic Viscosity Data for {[C_1C_2PIP][DMP] (1) + Ethanol (2)} Binary System as a Function of **(a)** Temperature for Different IL Mole Fraction, x_1: ♦, 1.0000; ♦, 0.8993; ◊, 0.7968; ■, 0.7031; ■, 0.6007; □, 0.5028; ▲, 0.4027; ▲, 0.3001; Δ, 0.2001; ●, 0.1001; ○, 0.0000. Points -Experimental Data; Solid Lines—Correlation Using Eq. (19) With Parameters Given in Table 11. **(b)** Composition at Different Temperature, T: ♦, 293.15 K; ◊, 303.15 K; ●, 313.15 K; ○, 323.15 K; ▲, 333.15 K. Points

Experimental Data; Solid Lines—guide to the eye.

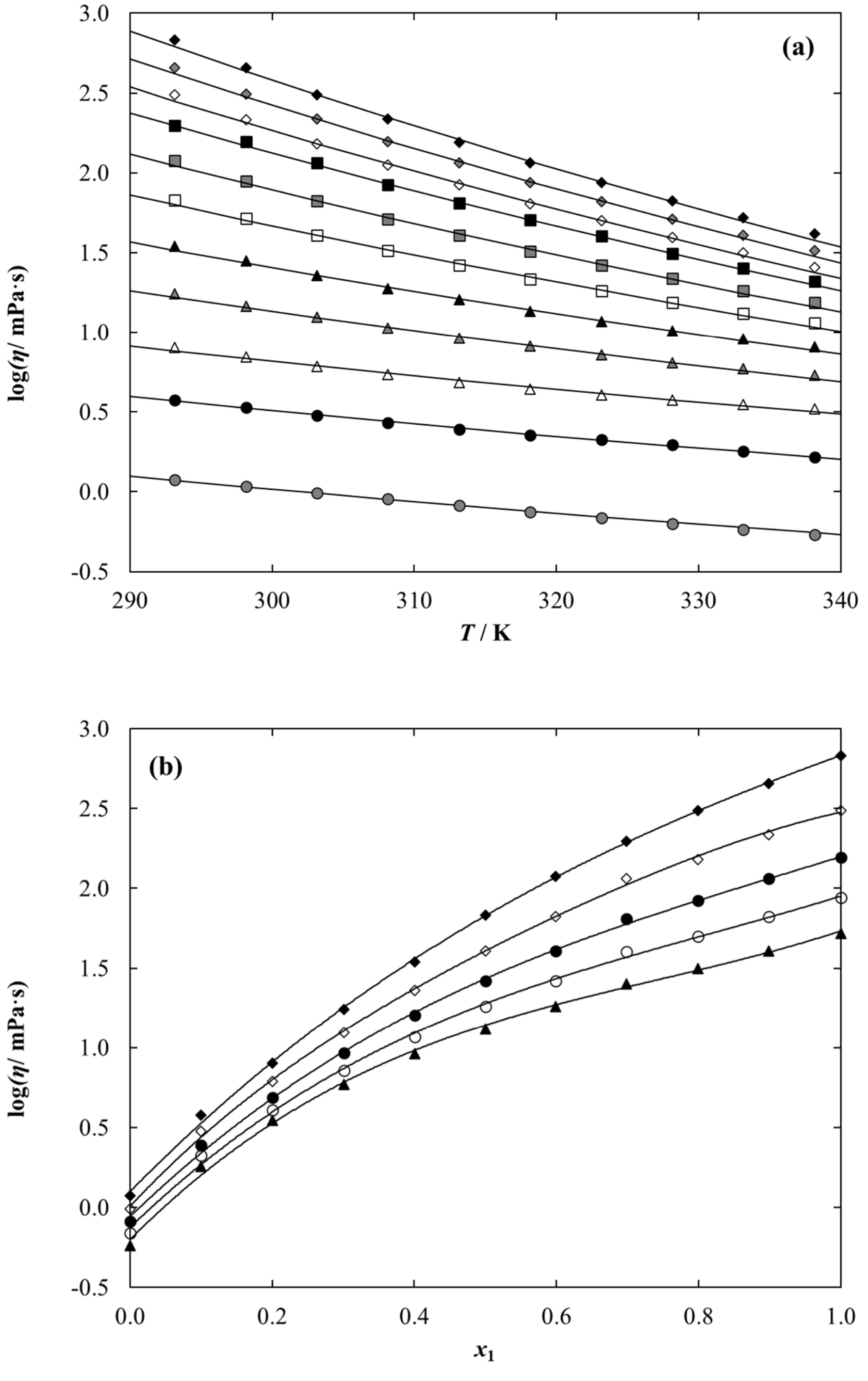

Figure S6. Experimental and Calculated Dynamic Viscosity Data for {[$N_{1,2,2,2}$][DMP] (1) + Ethanol (2)} Binary System as a Function of **(a)** Temperature for Different IL Mole Fraction, x_1: ♦, 1.0000; ♦, 0.8987; ◊, 0.7991; ■, 0.6988; ■, 0.5986; □, 0.4998; ▲, 0.4004; ▲, 0.3011; Δ, 0.1999; ●, 0.0998; ○, 0.0000. Points—Experimental Data; Solid Lines—Correlation Using Eq. (19) With Parameters Given in Table 11. **(b)** Composition at Different Temperature, T: ♦, 293.15 K; ◊, 303.15 K; ●, 313.15 K; ○, 323.15 K; ▲, 333.15 K. Points—Experimental Data; Solid Lines—guide to the eye.

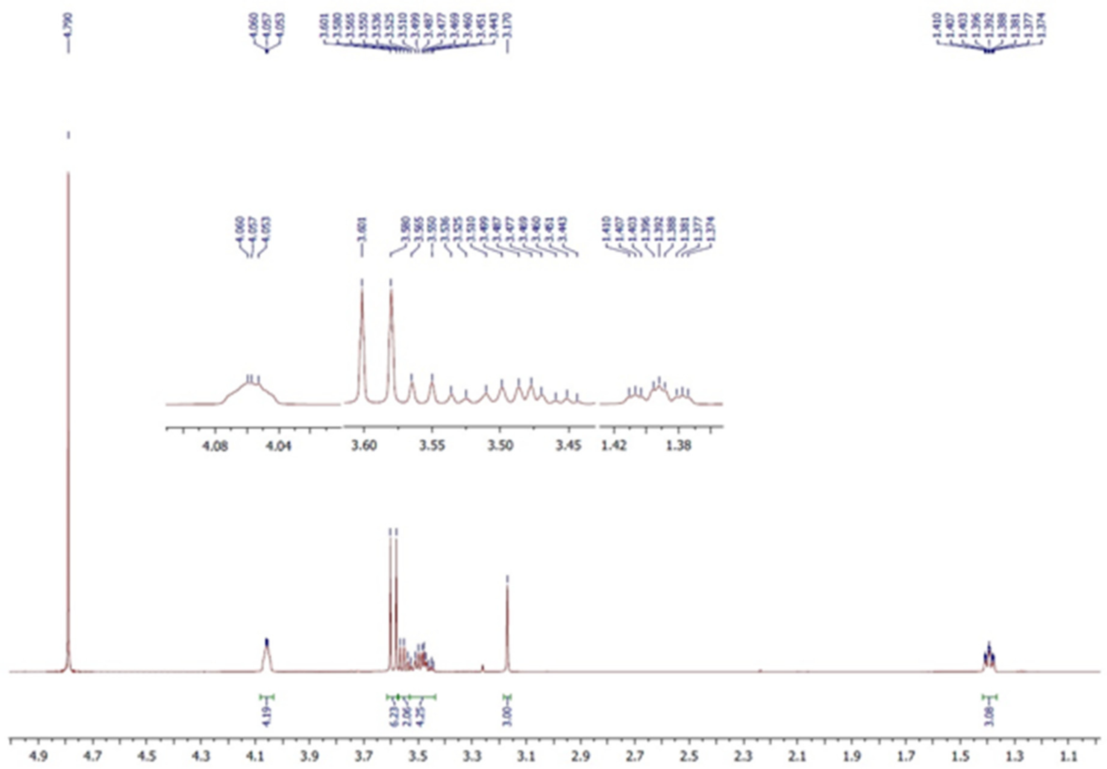

Figure S7. ^{1}H NMR Spectra of [C_1C_2MOR][DMP].

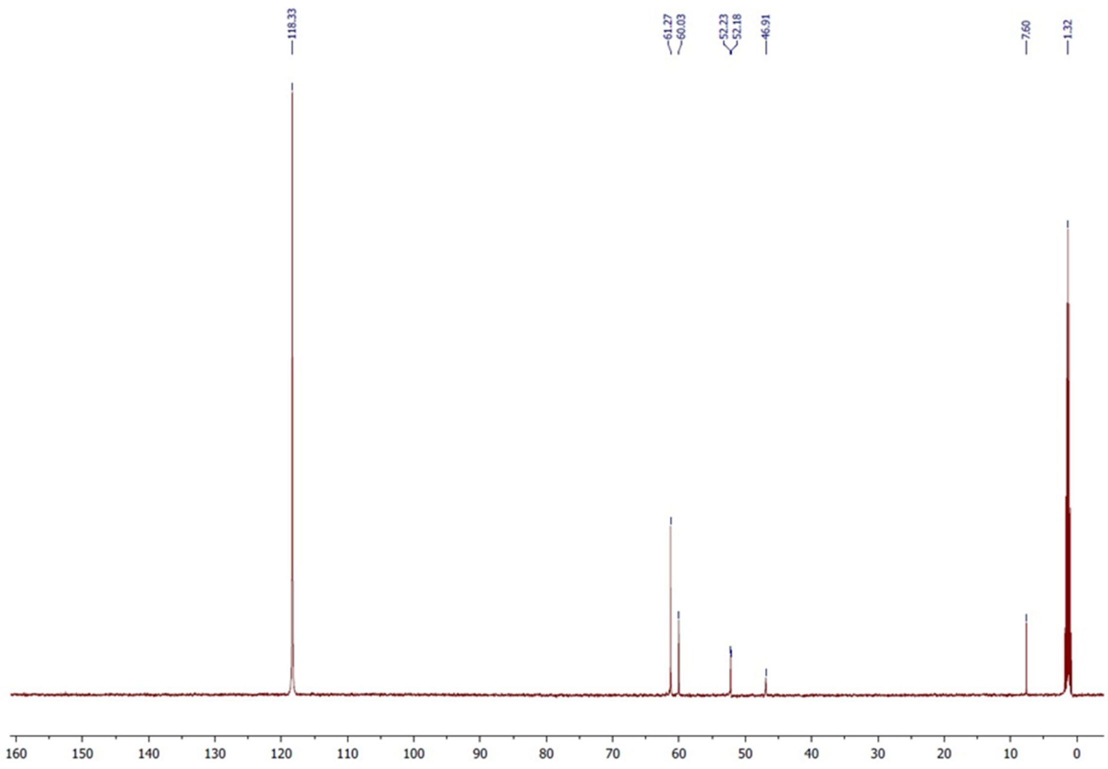

Figure S8. ^{13}C NMR Spectra of [C_1C_2MOR][DMP].
